# The OT-II model reveals dual *in vitro* and *in vivo* immunomodulatory properties of CD6 in T cell activation

**DOI:** 10.3389/fimmu.2025.1571590

**Published:** 2025-06-26

**Authors:** Alejandra Leyton-Pereira, Irene Fernández-Delgado, María José Rodríguez-Lagunas, Cristina Català, Sergi Casadó-Llombart, Noa Beatriz Martin-Cofreces, Eugenio Bustos-Morán, Natalia Díaz-Garrido, Marta Consuegra-Fernández, Ana Cristina Calpena, Fernando Aranda, María Velasco-de Andrés, Laura Baldomà, Francisco Sánchez-Madrid, Francisco Lozano

**Affiliations:** ^1^ Immunoreceptors del Sistema Innat i Adaptatiu, Institut d’Investigacions Biomèdiques August Pi i Sunyer, Barcelona, Spain; ^2^ Immunology Department, Instituto de Investigación Sanitaria Hospital Universitario La Princesa, Universidad Autónoma de Madrid, Madrid, Spain; ^3^ Departament de Bioquímica i Fisiologia, Facultat de Farmàcia i Ciències de l’Alimentació, Universitat de Barcelona, Barcelona, Spain; ^4^ Videomicroscopy Unit, Instituto de Investigación Sanitaria Hospital Universitario La Princesa, Madrid, Spain; ^5^ Centro de Investigación Biomédica en Red de Enfermedades Cardiovasculares (CIBERCV), Madrid, Spain; ^6^ Department of Pharmacy, Pharmaceutical Technology and Physical Chemistry, Faculty of Pharmacy and Food Sciences, University of Barcelona, Barcelona, Spain; ^7^ Institute of Nanoscience and Nanotechnology (IN2UB), University of Barcelona, Barcelona, Spain; ^8^ Servei d’Immunologia, Hospital Clínic de Barcelona, Barcelona, Spain; ^9^ Departament de Biomedicina, Universitat de Barcelona, Barcelona, Spain

**Keywords:** lymphocyte scavenger receptors, lymphocyte activation, CD6 deficiency, immunological synapse, immunomodulation, delayed T cell-mediated hypersensitivity

## Abstract

**Introduction:**

CD6 is a signal transducing transmembrane glycoprotein expressed on T-cells and a subset of B and NK cells that has emerged as a promising therapeutic target in autoimmunity and cancer.The extracellular domain of CD6 interacts with endogenous (CD166/ALCAM, Galectins 1 and 3,CD318/CDCP-1 and CD44) and exogenous (Pathogen-associated Molecular Patterns) ligands, and the phosphorylatable Thr/Ser/Tyr residues of its intracellular region can dock signal transduction effectors.This, together with its physical association with the T cell receptor (TCR) complex at the immunological synapse (IS) supports a relevant immunomodulatory role for CD6 in T cell activation , differentiation and survival. However, activation or inhibitory signalling properties have been observed by CD6 contingent on different experimental settings,rendering its precise function not well understood.

**Methods:**

To ascertain CD6’s immunomodulatory role, we investigated the effects of CD6-deficiency *in vitro* and *in vivo* under physiological antigen-specific stimulation in TCR transgenic OT-IImice.

**Results:**

*In vitro* ovalbumin (OVA)-specific stimulation of *Cd6* ^-/-^ OT-II splenocytes and *in vivo* OVA-induced delayed-type hypersensitivity (DTH) supported a negative modulatory role of CD6 in lymphocyte activation. On the contrary, IS studies in *Cd6* ^-/-^ OT-II T cells and OVA-loaded dendritic cells advocate for a positive modulatory role.

**Discussion:**

These findings support CD6 as a “dual” immunomodulatory receptor capable of either amplify or attenuate T-cell activation in different experimental contexts. This dual role should be taken into consideration while translating experimental data in to clinical applications, particularly in the development of CD6-targeted therapies for autoimmune disorders and cancer.

## Introduction

CD6 is a type I transmembrane glycoprotein of the scavenger receptor cysteine-rich (SRCR) superfamily relevant in T-cell activation, differentiation, and survival (1). This cell surface receptor is constitutively expressed on all T cells and a subset of B (B1a) and NK (CD56^lo^ CD16^hi^) cells, and active in various immune-mediated settings of clinical and therapeutic relevance (2). Recent genetic studies identified *CD6* variants as susceptibility or disease-modifying markers for autoimmune disorders (e.g., multiple sclerosis, inflammatory bowel disease, Behçet’s disease, psoriasis, and rheumatoid arthritis), prompting its evaluation as a potential therapeutic target (2–4).

CD6´s first and best studied ligand is CD166/ALCAM (Activated Leukocyte Cell Adhesion Molecule), an immunoglobulin superfamily cell adhesion molecule of wide tissue distribution (5). Other CD6 ligands include Galectins 1 and 3 (6), CD318/CDCP1 (Cub Domain Containing Protein 1) (7) and more recently CD44 (8). As for other SRCR superfamily receptors, CD6 also acts as pattern recognition receptor and interacts with pathogen-associated molecular patterns of bacterial, viral and parasitic origins (9, 10).

CD6 is physically associated with the clonotypic antigen-specific T-cell receptor (TCR) complex at the centre of the immune synapse (IS) (11, 12). CD6 lacks intrinsic catalytic activity, but its cytoplasmic tail can be phosphorylated by Ser/Thr and Tyr kinases and subsequently associate with signal-transduction effectors, positioning it as a potential modulator of key receptor signalling (13–15). Initially considered as a T-cell costimulatory molecule due to the co-mitogenic properties of certain anti-CD6 monoclonal antibodies (mAbs) (16–19), subsequent evidence from CD6 transfectants (20) and *Cd6* ^-/-^ mice (21, 22) also support a role for CD6 as an attenuator of TCR/CD3-mediated signalling. This immunomodulatory role is evidenced *in vivo* upon experimental induction of autoimmune T cell-mediated disease models in CD6-deficient mice. Thus, *Cd6* ^-/-^ mice show protection or attenuation in experimental autoimmune encephalomyelitis (EAE), autoimmune uveitis, and imiquimod-induced psoriasis models (21, 23, 24), while experience more severe collagen-induced arthritis (CIA), chronic graft-versus-host disease (cGvHD)-induced lupus-like disease, and antiviral humoral responses (22, 25, 26). These disparities highlight CD6’s complex role in T cell function under normal and pathological conditions.

The activating and inhibitory outcomes may result from experimental *in vitro* systems involving different cell types (e.g., normal or leukaemic T cells of human or rodent origin) and supraphysiological stimulation conditions (e.g., mAb-induced crosslinking, superantigens, adjuvants). To understand the biological role of CD6 in peripheral T cell immune regulation we have revisited the impact of CD6-deficiency *in vitro* and *in vivo*, on TCRαβ-transgenic OT-II mice under physiological antigen-specific stimulation, when peripheral CD4^+^ T lymphocytes recognise chicken ovalbumin (OVA)-specific sequences (OVA_323–339_) in the context of major histocompatibility complex (MHC) class II (H-2 I-A^b^) (27).

## Materials and methods

### Mice

CD6-deficient (*Cd6^-/-^*) were generated in C57BL/6N background as reported elsewhere (22). C57BL/6-Tg (TcraTcrb) 425Cbn/J mice expressing a T-cell receptor specific for the OVA_323–339_ peptide in the context of I-A^b^ (OT-II mice) (27) from the National Centre for Cardiovascular Research (CNIC; Madrid, Spain) were crossed with *Cd6^-/-^* mice to generate *Cd6^-/-^* OT-II mice and control *Cd6* ^+/+^ OT-II littermates. Sex-matched 8 to12 week-old mice were used in all the experiments. All mouse procedures were performed at the animal house of the University of Barcelona (UB) School of Medicine and approved by the UB-Animal Experimentation Ethical Committee (CEEA).

### Splenocyte stimulation assays

Single-cell splenocyte suspensions from *Cd6^-/-^* and *Cd6* ^+/+^ OT-II mice were obtained by mincing and passing spleens through a 40 μm nylon cell strainer (Biologix Research) using a syringe plunger. Following red blood cell lysis (eBioscience), cells were resuspended in complete RPMI-1640 medium (Invitrogen, Life Technologies) supplemented with 10% foetal bovine serum (FBS), 2 mM glutamine, 1 mM sodium pyruvate and 100 IU/mL penicillin/streptomycin and 50 μM of β-mercaptoethanol (Life Technologies). Splenocytes (1 x 10^5^ cells/well) were cultured in U-bottomed 96-well plates (VWR, North American) alone or in the presence of 1–10 µg/mL OVA_323–339_ peptide (ISQAVHAAHAEINEAGR, Sigma), 1–10 µg/mL OVA_257–264_ peptide (SIINFEKL, Sigma), 0.1–0.5 µg/mL anti-CD3ε mAb (145-2C11, TONBO biosciences) or 1 µg/mL anti-CD28 mAb (37.51, TONBO biosciences). Cultures were kept for different times at 37 °C in a 5% CO_2_ humidified atmosphere.

### T-APC conjugates assays

Spleen CD4^+^ T cells from OT-II *Cd6* ^-/-^ and OT-II *Cd6* ^+/+^ mice were isolated using a magnetic bead-based negative CD4^+^ isolation kit (STEMCELL Technologies, 19852) following the manufacturer´s instructions. In parallel, bone marrow cell progenitors obtained from C57/BL6 wild-type mice were used to generate bone-marrow derived dendritic cells (BMDCs) over 10 days in the presence of GM-CSF (20 ng/mL; PeproTech, 315-03). BMDCs were then matured for 24 h with LPS (250 ng/mL; Sigma-Aldrich, L2630) and pre-loaded or not with OVA_323–339_ peptide (5 μg/mL) for 2 h. Whole OVA protein (100 μg/mL; Sigma-Aldrich, A2512) was administered simultaneously with LPS to BMDCs. Subsequently, 1 μM 7-amino-4 chloromethylcoumarin (CMAC; Thermofisher Scientific) was added to BMDCs for immunofluorescence detection. CD4^+^ T cells and BMDCs were co-cultured (ratio 2:1) on poly-L-Lys-coated slides (50 μg/mL) for 1 h at 37 °C and then fixed for 10 min at room temperature (RT) with 4% paraformaldehyde (PFA). Cells were blocked and permeabilised for 30 min at RT in phosphate buffered saline (PBS) solution containing 3% bovine serum albumin (BSA), Fc-block (Tonbo Biosciences, 70-0161), 0.1% Triton X-100, 50 µg/mL human γ-globulin (Merck KGaA, G4386), 10% chicken serum (Merck KGaA, C5405) and 0.1% sodium azide. Afterwards, samples were stained with the following primary and secondary antibodies: rabbit anti-mouse CD3ζ 448 (kindly provided by B. Alarcon, Centro de Biología Molecular Severo Ochoa, Madrid), Alexa Fluor 647-labelled chicken anti-rabbit IgG (A-21443; Thermofisher Scientific), Alexa Fluor 568-labelled phalloidin (ThermoFisher Scientic A12380), and fluorescein isothiocyanate (FITC)-labelled anti-α-tubulin (clon DM1a, Sigma-Aldrich). Coverslips were mounted using Prolong Diamond (ThermoFisher Scientific P36965) and analysed with a Leica SP8 confocal microscope (Leica Microsystems, GmbH) fitted with a HCX PL APO 40x or 63x oil objective. Images were processed and assembled using Imaris and Photoshop software. The distance between the microtubule-organising centre (MTOC) and the cell-to-cell contact area was calculated using Imaris software; cell contact area was determined by coincidence of CMAC and CD3ζ signals for BMDCs and T cells, respectively.

### Western Blot analysis

Purified CD4^+^ T cells from OT-II *Cd6* ^-/-^ and OT-II *Cd6* ^+/+^ mice were co-cultured with mature BMDCs pre-loaded with OVA_323-339_ (5:1 ratio) for different times (0, 5 or 20 min). Cells were lysed for 20 min at 4 °C in a buffer containing 50 mM Tris-HCl pH 7.5 with 1% NP40, 0.2% Triton X-100, 150 mM NaCl, 2 mM EDTA, 1.5 mM MgCl_2_, and supplemented with phosphatase (PhosSTOP; Roche, 4906837001) and protease inhibitors (Complete; Roche, 11836145001). Cell lysate samples were mixed with Laemli’s solution and β-mercaptoethanol (final concentration 0.15 M) and boiled for 10 min at 100 °C, separated by SDS-PAGE and transferred to a nitrocellulose membrane. Membranes were blocked with Tris-buffered saline (TBS) containing 0.2% Tween 20 and 5% BSA then incubated overnight (o/n) at 4 °C with primary antibodies: anti-phospho-Y83 CD3ζ (Abcam; ab68236), anti-phospho-Y783 PLCγ (Cell Signalling Technologies; 2821), anti-phospho-T202/Y204 ERK 1/2 (Merck KGaA Calbiochem; SAB5701896) and anti-β-actin (Merck KGaA; clone AC15, A1978). After washing, membranes were incubated for 30 min at RT with peroxidase-labelled anti-rabbit Ig (ThermoFisher Scientifc 31460). ImageQuant LAS-4000 chemiluminescence and fluorescence imaging system (Fujifilm) was used for band detection. Band optical intensity was measured using ImageJ software and normalised to the loading control.

### Flow cytometry

Following splenocyte stimulation assays cells were blocked in PBS plus 2% FCS and anti-CD16/CD32 (2.4G2; Tonbo Biosciences) for 25 min at 4°C and then stained with the following fluorescent-labelled mAbs: APC-labelled rat anti-mouse Vα2 TCR (B20.1; BD Biosciences), PE-labelled anti-mouse CD6 (OX-129; BioLegend), APC-labelled anti-mouse CD3ε (145-2C11; Tonbo Biosciences), Pacific Blue-labelled anti-mouse CD4 (GK1.5; Biolegend), PE-labelled anti-mouse CD8α (53-6.7, BioLegend), APC-labelled anti-mouse CD25 (PC61.5; eBioscience) and eF450-labelled anti-mouse CD69 (H1.2F3; eBioscience). Staining of CD4^+^ T cells post conjugate formation (ratio 3:1) was done using the following primary antibodies: APC-labelled anti-mouse CD25 (PC61.5; Tonbo Biosciences), PECy7-labelled anti-mouse CD69 (H1.2F3; BD Biosciences), PerCP-labelled anti-mouse CD4 (RM4-5; Tonbo Biosciences), FITC-labelled anti-mouse CD44 (IM7; Tonbo Biosciences) and PE-labelled anti-mouse CD62L (MEL-14; BD Biosciences). A minimum of 20,000 events per sample were collected and analysed. Nonviable cells were excluded based on low forward and side scatter.

For proliferation assessment, total splenocytes were stained with 1 µM carboxyfluorescein-diacetate-succinimidyl-ester (CFSE), while isolated CD4^+^ T cells were stained with Cell Trace™ Violet according to the manufacturer’s instructions (Invitrogen). Dead cells were excluded using LIVE/DEAD Fixable Violet Dead Cell Staining dye (Invitrogen) or LIVE/DEAD Fixable Yellow Dead Cells Staining Dye (Invitrogen). Cell apoptosis was assessed using APC-Annexin V and 7-amino-actinomycin D (7AAD) staining set (BioLegend) following manufacturer’s instructions. All flow cytometry analyses were performed on a FACS Canto II equipped with CellQuest (BD Biosciences) and analysed using FlowJo V.10 software.

### Quantitative real-time PCR mRNA analyses

Total mRNA from splenocytes (1 x 10^6^ cells) stimulated with OVA_323–339_ peptide (5 µg/mL) or soluble anti-CD3ε mAb (0.5 µg/mL) for 24 h was extracted using the β-mercaptoethanol and the PureLink™ RNA Mini Kit (Ambion, Life Technologies) following manufacturer’s instructions. RNA was quantified and retrotranscribed into cDNA by using the High-capacity cDNA Kit (Life Technologies). mRNA levels were assessed by qRT-PCR using the TaqMan™ Fast Universal PCR Master Mix No AmpErase™ UNG (Life Technologies) on a 7900HT fast real-time PCR system (Applied Biosystems). The following TaqMan probes were used: Mm00801778_m1 (*Ifng*), Mm00434256_m1 (*Il2*), Mm01204974_m1 (*Fas*), Mm00438864_m1 (*Fasl*), Mm00435532_m1 (*Pd1*) and Mm00452054_m1 (*Pdl1*), all from ThermoFisher Scientific. Relative mRNA expression was normalised to *Gapdh* expression and calculated using the 2-ΔCt method, where ΔCt = (CT_Gene_ − CT*_Gapdh_*).

Total RNA from loco regional lymph nodes was isolated using the TRIzol method (ThermoFisher Scientific). Tissue was homogenized in 1 mL of ice-cold TRI Reagent^®^ (Sigma Aldrich) using a Polytron™ Homogenizer PT1200E (Thermo Fisher Scientific) for 4 min. RNA was extracted following the manufacturer’s instructions, and its concentration and quality were assessed using a NanoDrop™ 2000/2000c Spectrophotometer (ThermoFisher Scientific). RNA integrity was verified by visualising 28S and 18S rRNA via agarose/formaldehyde gel electrophoresis. RNA (1 µg) was reverse transcribed into cDNA using the High Capacity cDNA Reverse Transcription kit (Applied Biosystems) in a final volume of 20 µL. RT-qPCR was performed using a StepOne Plus PCR cycler (Applied Biosystems) with SYBR^®^ Green PCR Master Mix (Applied Biosystems) and forward (Fw) and reverse (Rv) oligonucleotide probes specific for *Ifng* (Fw: TGAAAGACAATCAGGCCATC; Rv: 5’-TTGCTGTTGCTGAAGAAGGT-3’), *Il4* (Fw: 5’-TCAACCCCCAGCTAGTTGTC-3’; Rv: 5’-TGTTCTTCGTTGCTGTGAGG-3’), *Il7a* (Fw: 5’-ATCAGGACGCGCAAACATGA-3’; Rv: 5’-TTGGACACGCTGAGCTTTGA-3’), *Tbx21* (Fw: 5’-CCTGGACCCAACTGTCAACT-3; Rv: 5’-AACTGTGTTCCCGAGGTGTC-3’), *Rorgc* (Fw: 5’-GACAGGGAGCCAAGTTCTCAG-3’; Rv: 5’-TCGGTCAATGGGGCAGTTC-3’), *Gata3* (Fw: 5’-GAACCGCCCCTTATCAAG-3’; Rv: 5’-CAGGATGTCCCTGCTCTCCTT-3’), *Gadph* (Fw: 5’-AACTTTGGCATTGTGGAAGG-3’; Rv: 5’-ACACATTGGGGGTAGGAACA-3’), and *Hptr* (Fw: 5’-TCCTCCTCAGACCGCTTTT-3’; Rv: 5’-CCTGGTTCATCATCGCTAATC-3’). Cycling conditions were: 1 cycle of 10 min at 95°C, followed by 40 cycles of 15 s at 95°C and 1 min at 60°C. Relative gene expression was normalised to *Hptr* and *Gapdh*. The 2-ΔCt method was used to calculate fold-change.

### ELISA assays

Mouse IL-2 and IFN-γ cytokine levels in cell culture supernatants were measured by using BD OptEIA sets (BD Biosciences) following manufacturer’s instructions.

### Delayed-type hypersensitivity model

*Cd6* ^-/-^ and *Cd6* ^+/+^ OT-II mice were sensitized two consecutive days by intradermal (i.d.) injection of 100 μg EndoGrade^®^ OVA (Hyglos, Germany) diluted in 200 μl of pyrogen-free 0.9% saline (Braun) without adjuvants. The solution was divided and injected into four sites on the alcohol-cleaned, unshaved abdominal skin. On day 4 post-sensitisation, ear swelling was elicited under isoflurane anaesthesia (Abbott Laboratories, Berkshire, UK) and 10 µl containing 5 μg of OVA in saline (OVA-OVA) were injected (i.d.) into both ears. Control mice were sensitized with saline alone and challenged with either OVA (PBS-OVA) or saline (PBS-PBS). Ear thickness was measured using a micrometre (Neoteck Company, China) before and 24 h after challenge, expressed as mean (mm) ± SE. Ear swelling was also quantified by analysing weight differences (in mg) between 4-mm discs obtained from treated and untreated ears. For histological analyses, ears and loco regional lymph nodes were excised immediately after sacrifice and fixed o.n. in 4% buffered formaldehyde at RT. Samples were then washed in PBS for 3 h, dehydrated in graded ethanol (70%, 90%, and 100%), permeated with xylene, and embedded in paraffin wax. Sections (5 μm) were cut using a Leica HistoCore MULTICUT (Germany), stained with haematoxylin and eosin (H&E), and analysed using an Olympus BX41 (Japan) bright-field microscope and an Olympus XC50 camera (Japan) at 100x or 200x magnification. Immunohistochemical staining was performed using the HRP/DAB rabbit-specific detection IHC kit (ab64261, Abcam, Victoria, Australia). Briefly, 5 μm paraffin-embedded sections were dewaxed in xylene, rehydrated in a series of graded ethanol (100%, 100%, 95% 70% and 50%), and subjected to antigen retrieval in citrate buffer (pH 6) at 121°C for 10 min. After the washing and blocking steps, slides were incubated o.n. at 4°C with rabbit polyclonal anti-IL-22 (1:500; Invitrogen, PA121357) and anti-IFN-γ (1:500, ThermoFisher Scientific, PA595560) antisera. Secondary antibody incubation and DAB staining were performed according to manufacturer’s instructions. Negative controls omitted the primary antibody. Images were captured using an Olympus BX41 microscope and Olympus XC50 camera at 20x, 40x, and 60x magnification and analysed using ImageJ software (NIH Image, Bethesda, MD, USA).

### Statistical analyses

Data were expressed as mean ± SEM. Statistical analyses were performed using parametric t-tests for paired data using GraphPad Prism (GraphPad Software).

## Results

### CD6 deficiency up-regulates antigen-specific T cell activation and proliferation without impacting apoptosis

**Figure 1:**
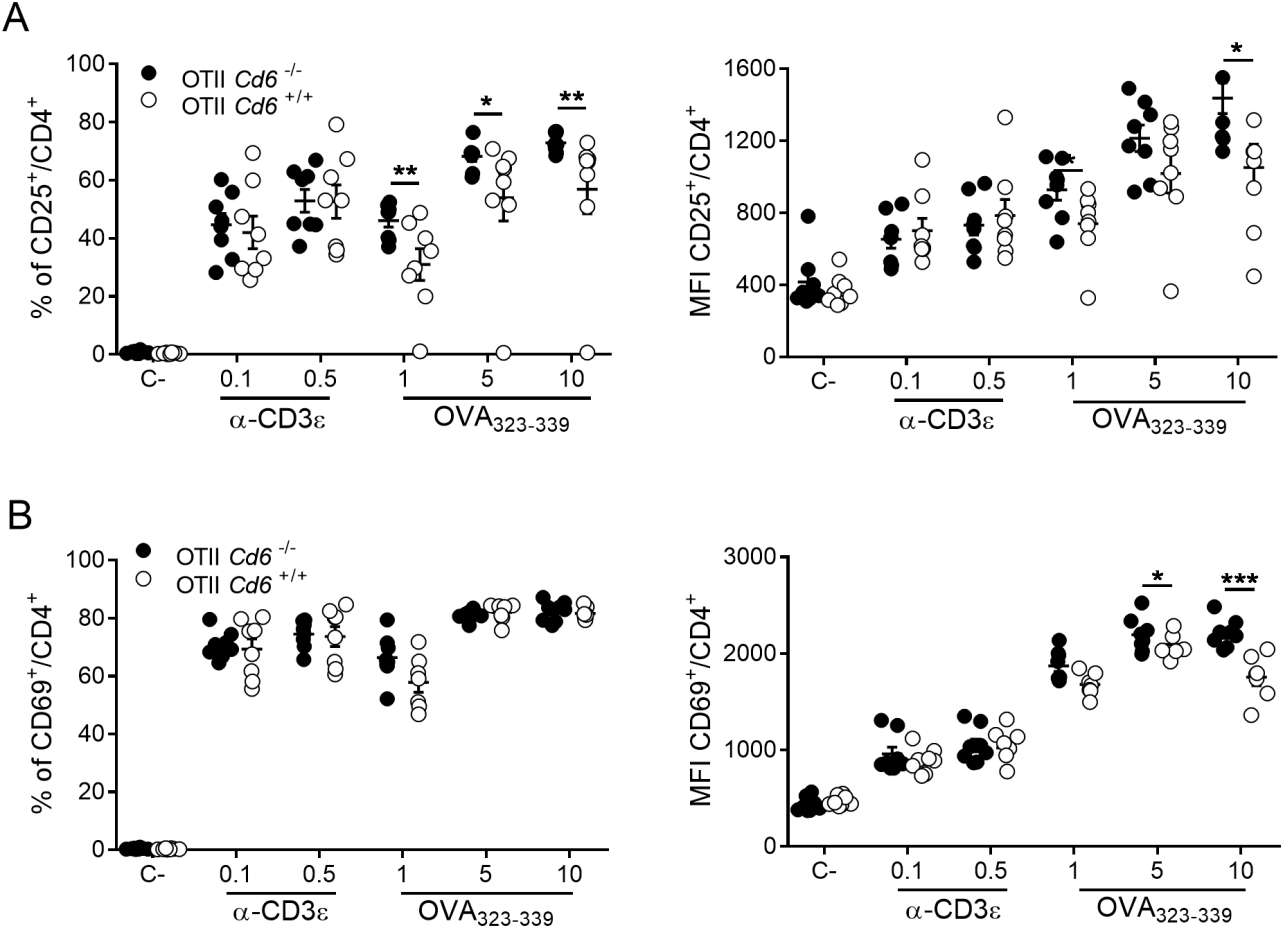
Antigen-specific activation increases surface CD25 and CD69 expression of OT-II *Cd6^-/-^* CD4^+^ T-cells. Splenocytes from OT-II *Cd6^-/-^* or OT-II *Cd6^+/+^* mice were exposed for 24 h to OVA_323-339_. In parallel, OVA_257-264_ (C-, 10 µg/mL) and soluble α-CD3ϵ mAb were included as negative and positive controls, respectively. Percentage (left) and MFI (right) of CD25^+^ **(A)** and CD69^+^ **(B)** gated CD4^+^ T-cells were assessed by flow cytometry. Data are mean ± SEM values. A representative experiment out of three is shown. Statistical differences were assessed by unpaired Student’s t test and Mann-Whitney test. ∗*p* < 0.05; ∗∗*p* < 0.01; ∗∗∗*p* < 0.001.

The immunomodulatory properties of CD6 expression following physiological antigen-specific stimulation of peripheral T lymphocytes were first assessed by exposing *Cd6* ^-/-^ and *Cd6* ^+/+^ OT-II splenocytes to increasing concentrations of OVA_323–339_ peptide (1-10 µg/mL) for 24 h. MHC class I-restricted OVA_257–264_ and mAb-induced CD3ϵ crosslinking were used as negative and positive controls, respectively. In the latter, suboptimal α-CD3ϵ (145-2C11) mAb concentrations (0.1 and 0.5 µg/mL) were used to prevent overshadowing by maximal activation signal strength of potential CD6-mediated positive or negative modulatory effects. As illustrated by **Figure 1**, flow cytometry analyses of OVA_323–339_ challenged *Cd6* ^-/-^ OT-II CD4^+^ T cells showed higher surface expression levels (measured by Median Fluorescence Intensity; MFI) of the early T cell activation markers CD69 and CD25. No such differences were observed following mAb-induced CD3 crosslinking conditions. This observation indicates that CD6 expression fine-tunes early CD4^+^ T cell activation events by attenuating TCR signalling following antigen-specific recognition.

**Figure 2:**
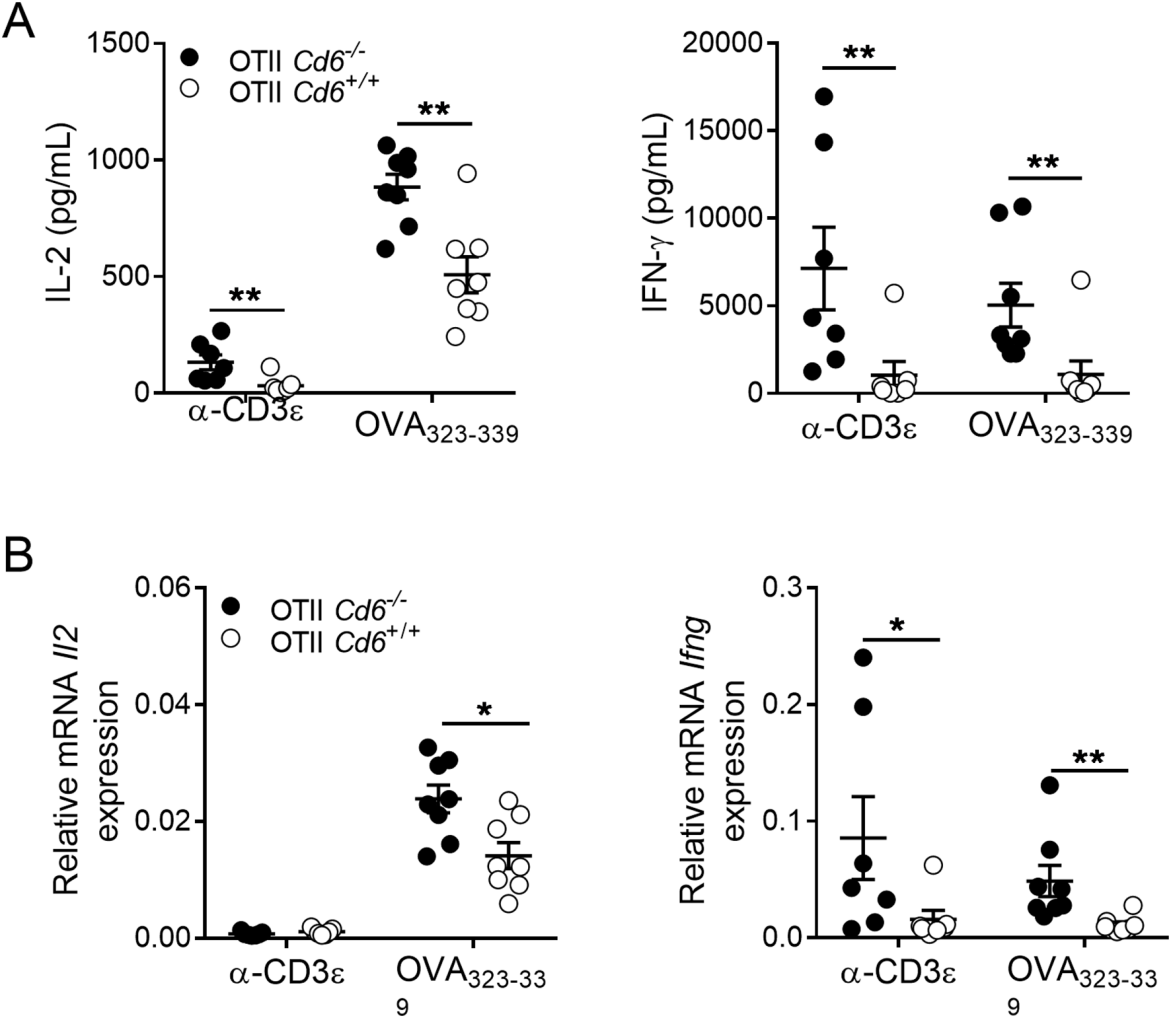
Antigen-specific activation increases IL-2 and IFN-γ expression of OT-II *Cd6^-/-^* splenocytes. **(A)** Culture supernatants from *Cd6^-/-^* and *Cd6^+/+^* OT-II splenocytes stimulated for 24 h with α-CD3ϵ mAb (0.5 μg/mL) or OVA_323-339_ (5 μg/mL) were assayed for IL-2 and IFN-γ levels by ELISA. A representative experiment out of two performed is shown. Statistical differences were assessed by Mann-Whitney test. ∗∗, *p* < 0.01. **(B)** *Il2* and *Ifng* mRNA expression levels from the same cell cultures as in **(A)** were analysed by qRT-PCR. Relative mRNA expression indicates the difference in the threshold cycle between *Gadph* and target genes. Data are mean ± SEM values. A representative experiment out of two performed is shown. Statistical differences were assessed by Mann-Whitney test. ∗*p* < 0.05; ∗∗*p* < 0.01.

Further support of this observation also came from cytokine studies. As illustrated in **Figure 2A**, ELISA analysis of culture supernatants from OVA_323-339_-challenged (5 μg/mL) *Cd6* ^-/-^ and *Cd6* ^+/+^ OT-II splenocytes for 24 h revealed higher levels of IL-2 and IFN-γ for *Cd6* ^-/-^ OT-II splenocytes. RT-qPCR analysis further confirmed higher relative levels of *Il2* and *Ifng* mRNA in *Cd6* ^-/-^ OT-II splenocytes (**Figure 2B**), suggesting increased synthesis rather than decreased consumption. A similar observation was made for *Cd6* ^-/-^ OT-II splenocytes subjected to suboptimal mAb-induced CD3 crosslinking (0.5 μg/mL), except for *Il2* mRNA levels.

**Figure 3:**
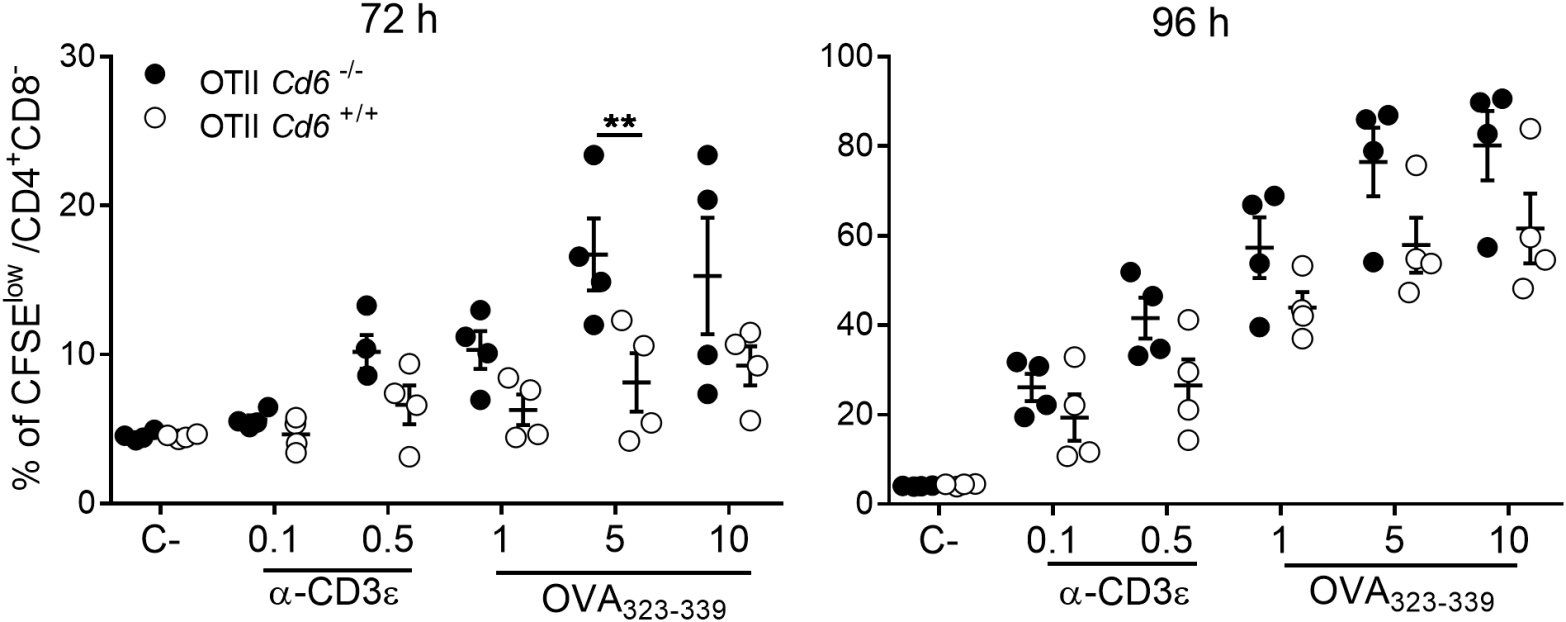
Antigen-specific activation increases proliferative responses of OT-II *Cd6^-/-^* splenocytes. CFSE-labelled *Cd6^-/-^* and *Cd6^+/+^* OT-II splenocytes were exposed to OVA_257-264_ (C-, 10 µg/mL), soluble α-CD3ϵ mAb and OVA_323–339_ for 72 and 96 h. Percent of CFSE^low^ CD4^+^ T cells is shown, as assessed by flow cytometry at 72 h (left) and 96 h (right). Data are mean ± SEM values. Graphs show a representative experiment out of three performed. Statistical differences were assessed by ANOVA two-way ∗∗*p* < 0.001.

To test whether CD6 expression also modulates antigen-specific lymphoproliferative responses, CFSE-stained *Cd6* ^-/-^ and *Cd6* ^+/+^ OT-II splenocytes were exposed to OVA_323-339_ (1, 5 and 10 μg/mL) for 72–96 h, using OVA_257-264_ (10 µg/mL) and mAb-induced CD3ϵ crosslinking (0.1 and 0.5 μg/mL) as negative and positive controls, respectively. As illustrated in **Figure 3**, C*d6* ^-/-^ OT-II splenocytes exhibited a higher percentage of CFSE^low^ CD4^+^ T cells upon stimulation with OVA_323–339_ compared to *Cd6* ^+/+^ OT-II, reaching statistical significance at 72 h. No differences were observed between both study groups following mAb-induced CD3 crosslinking. Collectively, the findings support a down-modulatory role of CD6 expression on activation and proliferation of CD4^+^ T cells under antigen-specific stimulatory conditions.

**Figure 4:**
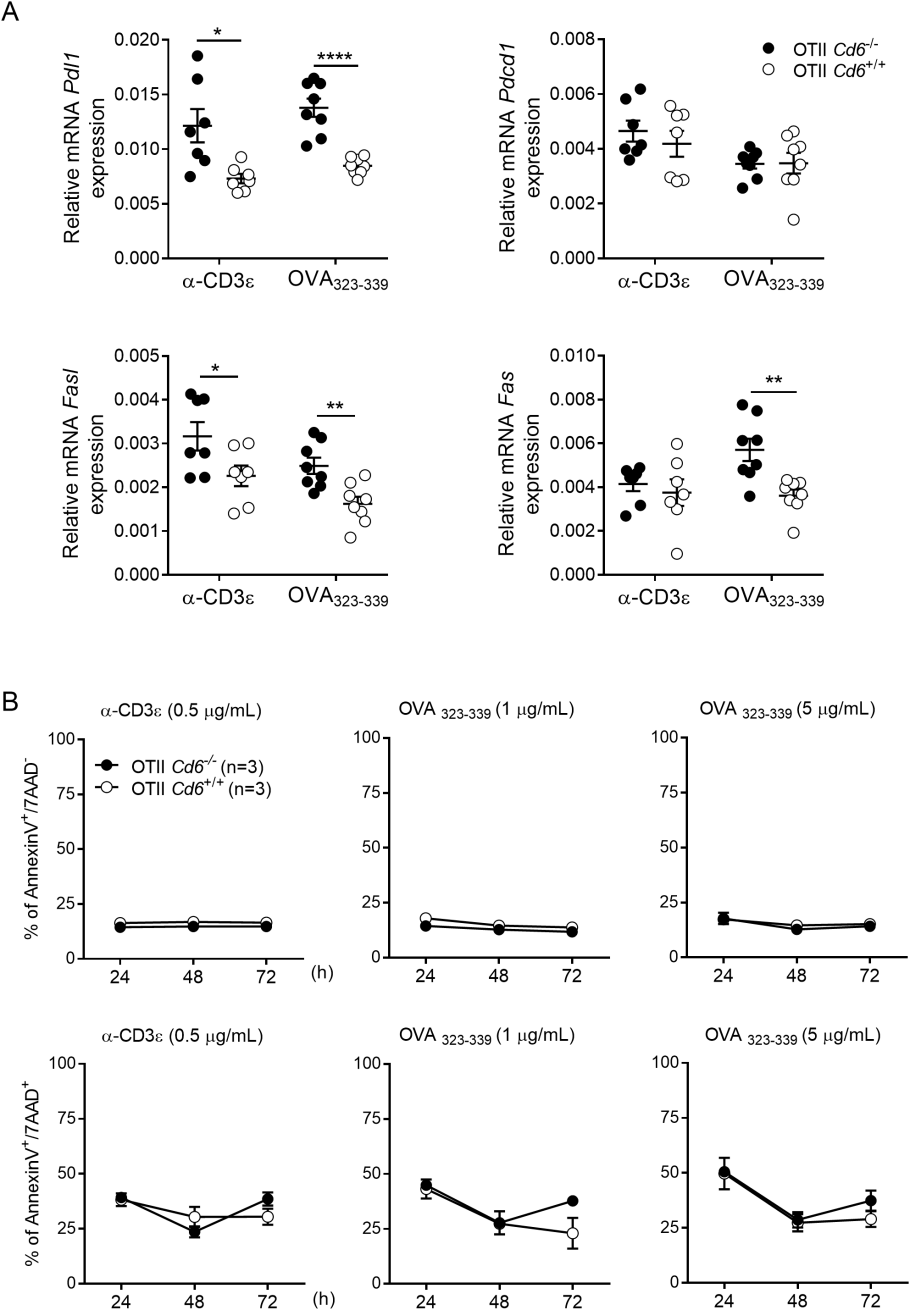
Antigen-specific activation of OT-II *Cd6^-/-^* splenocytes increases the expression of AICD markers without impacting cell apoptosis. **(A)** Relative *Pdl1*, *Pdcd1*, *Fasl* and *Fas* mRNA expression levels from *Cd6^-/-^* and *Cd6^+/+^* OT-II splenocytes stimulated for 24 h with α-CD3ϵ mAb (0.5 μg/mL) or OVA_323-339_ (5 μg/mL), as analysed by qRT-PCR. Relative mRNA expression indicates the difference in the threshold cycle between *Gapdh* and target genes. Data with error bars are presented as the mean ± SEM values. Statistical differences were assessed by unpaired Student’s t test. ∗*p* < 0.05; ∗∗*p* < 0.01, ∗∗∗∗*p* < 0.0001. **(B)** *Cd6^-/-^* or *Cd6^+/+^* OT-II splenocytes were stimulated for 24, 48 and 72 h with α-CD3ϵ mAb (0.5 μg/mL) or OVA_323-339_ (1 and 5 μg/mL). Then, percent of apoptotic (annexin V^+^ 7AAD^-^) and late apoptotic/necrotic cells (annexin V^+^ 7AAD^+^) cells was assessed on gated CD4^+^ T cells by flow cytometry. Data with error bars are presented as the mean ± SEM values. A representative experiment out of five performed is shown. Statistical differences were assessed by ANOVA two-way.

Increased activation signals delivered by the TCR/CD3 complex upon specific antigen recognition may lead to activation-induced cell death (AICD), depending on the expression of different AICD markers such as Fas, FasL, PD-1 and PD-L1. Thus, we further analysed relative mRNA expression of these surface AICD markers in *Cd6* ^-/-^ and *Cd6* ^+/+^ OT-II splenocytes stimulated with α-CD3ϵ mAb (0.5 μg/mL) or OVA_323-339_ (5 μg/mL) for 24 h. As illustrated in **Figure 4A**, qRT-PCR analyses revealed increased *Pdl1*, *Fas*, and *Fasl* mRNA levels upon OVA_323–339_ exposure to *Cd6* ^-/-^ OT-II splenocytes, and of *Pdl1* and *Fasl* upon mAb-induced CD3 crosslinking, indicating that *Cd6* ^-/-^ OT-II T cells can be more prone to undergo AICD upon a further stimulation. Annexin V and 7AAD staining showed no apoptosis differences between *Cd6* ^-/-^ and *Cd6* ^+/+^ OT-II splenocytes upon a first challenge with antigen-specific and mAb-induced CD3 crosslinking stimulation (**Figure 4B**). This indicates that increased lymphocyte activation associated to CD6 deficiency may help increase AICD.

### CD6 deficiency exacerbates OVA-induced DTH

**Figure 5:**
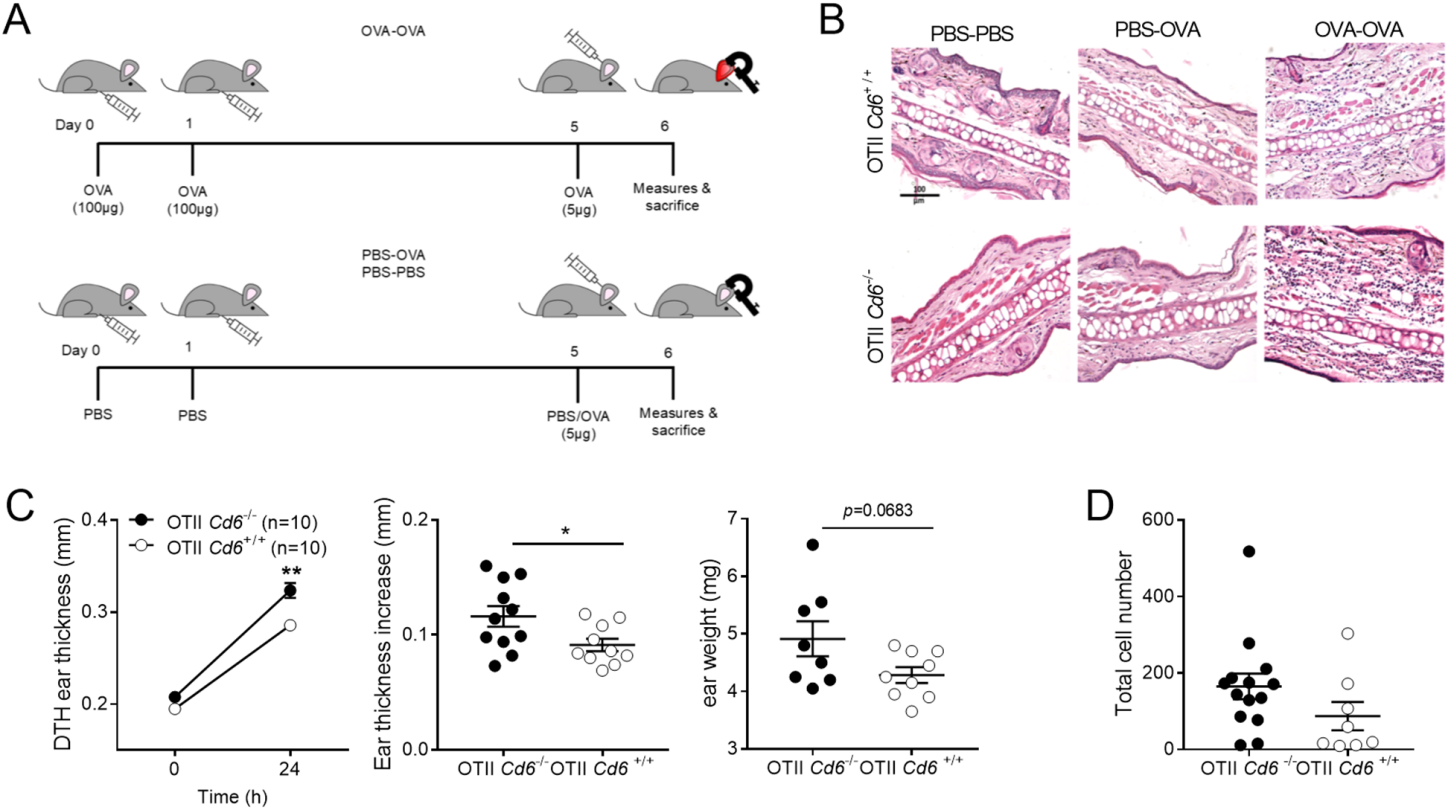
CD6 deficiency exacerbates OVA-induced DTH. **(A)** Schematic representation of OVA-DTH sensitisation and induction groups analysed. **(B)** Representative H&E staining of 5 µm ear sections of the different groups analysed. Bar = 100 µm (200x). **(C)** Ear thickness (mm) and weight (mg) from OT-II *Cd6* ^-/-^ and OT-II *Cd6* ^+/+^ mice of the OVA-OVA group before and 24 h post DTH-induction is shown. Data are mean ± SEM. A representative experiment out of three performed is shown. Statistical differences were assessed by unpaired Student’s *t* test. ∗*p*  <  0.05; ∗∗*p*  <  0.01. **(D)** Total polymorphonuclear and lymphocyte cell counts in H&E sections from OVA-OVA group. The PBS-PBS and PBS-OVA groups rendered very low cell counts (not shown). Data (mean ± SEM) are representative of one out of three individual experiments performed, with n = 8–14 technical replicates per genotype per experiment. Statistical differences were assessed by unpaired Student’s *t* test.

As an alternative approach to validate our *in vitro* data, the immunomodulatory role of CD6 in antigen-specific CD4^+^ T cell responses was investigated by the OVA-induced DTH model in *Cd6* ^-/-^ and *Cd6* ^+/+^ OT-II mice (28). The protocol entailed sensitisation of mice by intradermal administration of OVA (100 µg) in the abdominal area for two consecutive days, followed by DTH induction by a further OVA challenge (5 μg) in both ears four days later (OVA-OVA group). Negative control groups were included, in which OVA sensitisation and/or DTH induction was omitted (PBS-PBS and PBS/OVA, respectively) (**Figure 5A**). As is illustrated in **Figure 5B**, H&E staining revealed increased ear oedema and cellular infiltration in the OVA-OVA groups compared to the control PBS-PBS and PBS-OVA groups. Further analyses revealed that *Cd6* ^-/-^ OT-II mice exhibited greater ear thickness as well as weight (4-mm ear discs) at 24 h post DTH induction compared to *Cd6* ^+/+^ OT-II (**Figure 5C**). However, there were non-significant differences in the number of infiltrating neutrophils and lymphocytes between *Cd6* ^-/-^ and *Cd6* ^+/+^ OT-II OVA-OVA groups (**Figure 5D**).

**Figure 6:**
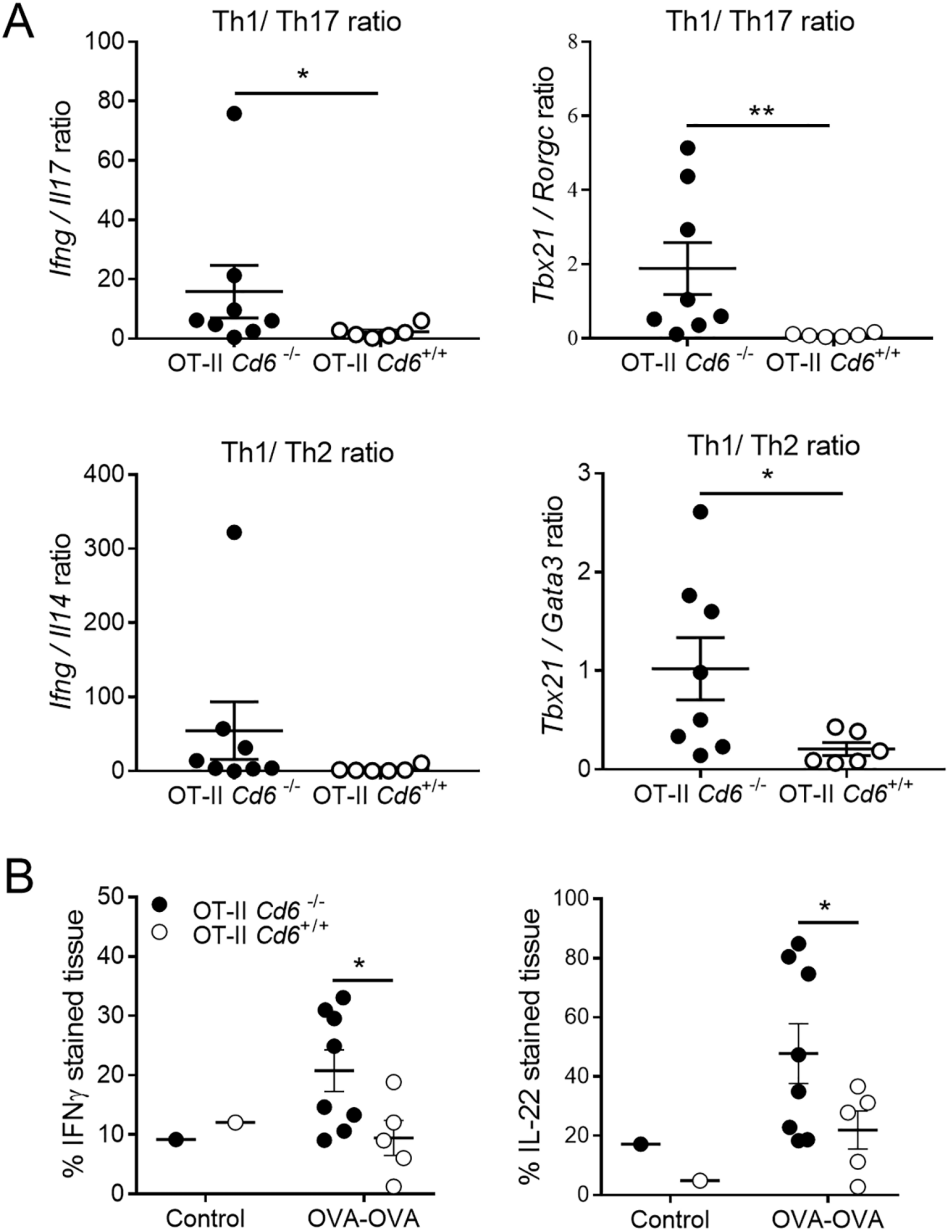
Cytokine mRNA and protein expression analyses in lymph node from OT-II *Cd6^-/-^* or OT-II *Cd6^+/+^* mice undergoing OVA-induced DTH. **(A)** Reversed transcribed total RNA from *Cd6^-/-^* or *Cd6^+/+^* OT-II locoregional lymph nodes of the OVA-OVA group were subjected to RT-qPCR analysis of the indicated cytokine and transcription factor genes. Results are mean ± SEM values for Th1/Th17 and Th1/Th2 ratios. Relative mRNA expression indicates the difference in the threshold cycle between *Gadph* and *Hptr-1* and target genes. Data are from one individual experiment with n = 6–8 technical replicates per genotype and per experiment. Statistical differences were assessed by unpaired Student’s *t* test. ∗*p* < 0.05; ∗∗*p* < 0.01. **(B)** Immunohistochemical analyses of locoregional lymph node sections from *Cd6^-/-^* or *Cd6^+/+^* OT-II mice of the OVA-OVA and Control (PBS/PBS and PBS/OVA) groups collected at 24 h post OVA-DTH induction. Data are mean ± SEM of the percentage of IFN-γ- and IL-22-stained tissue. Statistical differences were assessed by unpaired Student’s *t* test. ∗*p* < 0.05.

The expression analysis of Th1, Th2 and Th17 cytokines (*Ifng*, *Il4* and *Il17*) and transcription factor (*Tbx21*, *Gata3* and *Rorgc*) genes in regional lymph nodes at 24 post OVA-DTH induction revealed higher Th1/Th17 (*Ifng*/*Il17*, *Tbx21*/*Rorgc*) and Th1/Th2 ratios (*Ifng*/*Il4*, *Tbx21*/*Gata3*) for *Cd6* ^-/-^ OT-II mice compared to *Cd6* ^+/+^ OT-II (**Figure 6A**). Further immunohistochemistry analysis of locoregional lymph nodes also showed increased percentages of IFN-γ- and IL-22-positive stained tissue for *Cd6* ^-/-^ OT-II mice compared to *Cd6* ^+/+^ OT-II (**Figure 6B**). These findings indicate that CD6 deficiency associates with exacerbated *in vivo* Th1-type response in the OVA-induced DTH model, an observation that aligns with the negative modulatory function of CD6 observed during *in vitro* OVA antigen-specific activation of *Cd6* ^-/-^ OT-II splenocytes.

### CD6 deficiency attenuates T-APC immune synapse formation and subsequent signalling and cell proliferation

**Figure 7:**
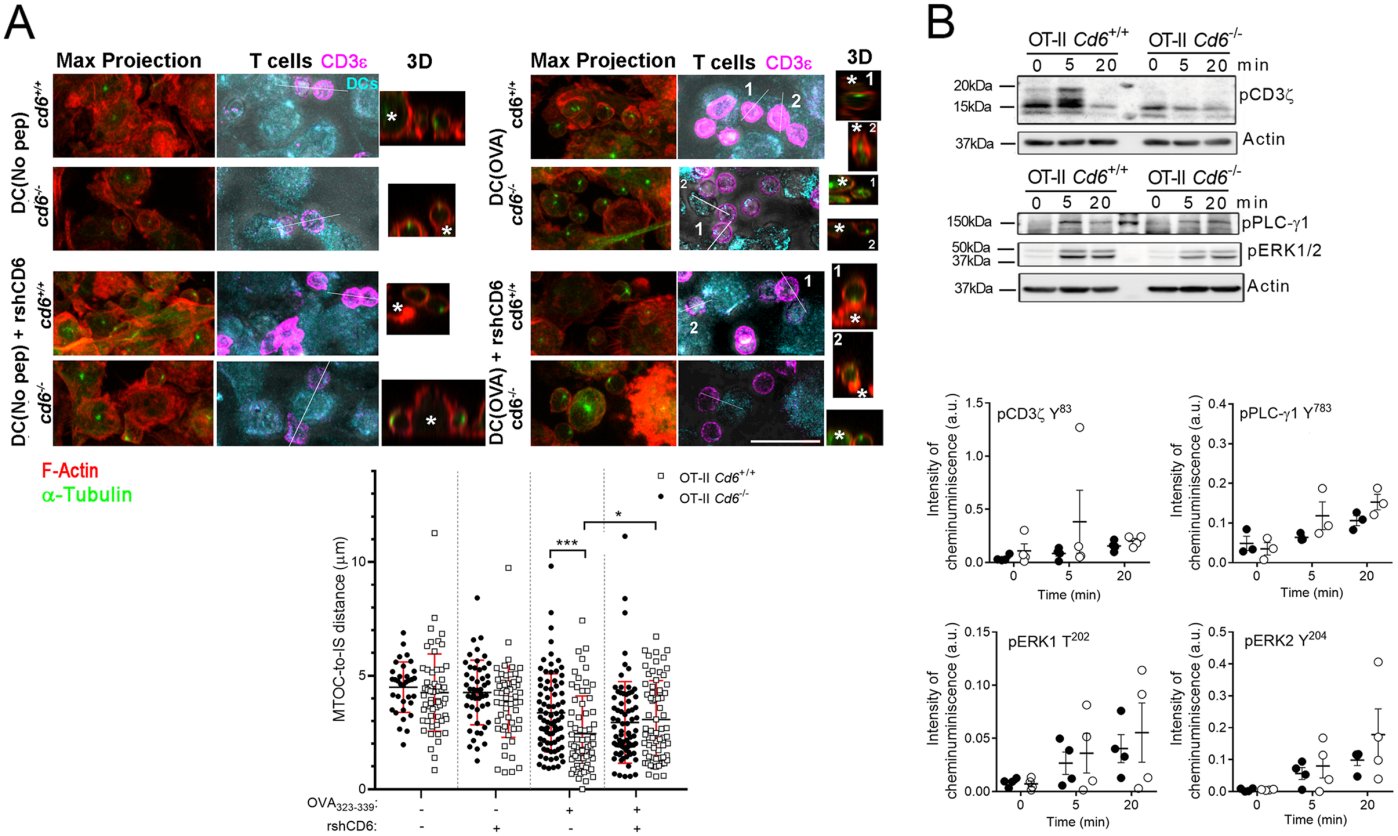
CD6 deficiency negatively regulates IS formation and subsequent early intracellular TCR signalling. **(A)** OVA_323-339_-loaded mature BMDCs labelled with CMAC (cyan) were co-incubated for 1 h with CD4^+^ T cell from *Cd6^-/-^* or *Cd6^+/+^* OT-II spleens in the presence (+) or absence (–) of rshCD6 (10 µg/mL). Samples were fixed and stained with specific anti-α-tubulin-FITC to detect MTOC orientation, as well as with Alexa-568-labelled phalloidin (red) and Alexa-647-labelled anti-CD3ζ (magenta). The distance of the MTOC to the T-BMDC contact zone was quantified with Imaris, relative to CD3ζ and CMAC coincidence. A representative image of each group is shown (top). Maximal projections are included; bright field is shown together with CMAC and CD3ζ for localisation of cells. 3D reconstruction of the indicated T cells are shown. Asterisks indicate the localisation of the BMDC. Bar, 20 µm. Data are mean ± SD values from two independent experiments with error bars (bottom). Statistical differences were assessed by ANOVA one-way. *p < 0.05; ***p < 0.001. **(B)** Western blot analysis of lysates from conjugates of OVA_323-339_-loaded BMDCs and *Cd6^-/-^* or *Cd6^+/+^* OT-II CD4^+^ T cells (ratio 5:1). Phosphorylation for CD3ζ-Y^83^, PLCγ-Y^783^ and ERK 1/2-T^202^/Y^204^ for the indicated times (top) are shown. Quantification of the relative band intensity is shown as mean ± SD (bottom). Statistical differences were assessed by unpaired Student’s t test and Mann-Whitney test.

The results *in vitro* and *in vivo* from the OT-II model advocate for a negative immunomodulatory function of CD6. This contrasts previous independent reports showing that CD6-CD166/ALCAM interaction positively impacts T-APC conjugate formation and subsequent T-cell proliferation (11, 12). As *Cd6* ^-/-^ mice were not available at the time, such experiments conjugated primary (polyclonal SEB-specific) or leukaemic (Jurkat) human T cells with superantigen (SEE or SEB)-loaded APCs (monocyte-derived DCs or Raji B cells), in the presence or absence of specific blocking agents (soluble human recombinant CD6 or ALCAM proteins). On the basis of such diverse experimental procedures, we revisited IS studies by performing antigen-specific T-APC conjugates formation involving OVA_323-339_-loaded mature OT-II BMDCs and *Cd6* ^-/-^ and *Cd6* ^+/+^ OT-II CD4^+^ T cells in the presence or absence of recombinant soluble human CD6 protein (rshCD6), known to have interspecies binding to both mouse and human CD166/ALCAM (29). As illustrated in **Figure 7A**, activated *Cd6* ^+/+^ OT-II T cells exhibited reduced distance of MTOC-to-IS compared to *Cd6* ^-/-^ OT-II ones. In the presence of rshCD6, the MTOC-to-IS distance increased in *Cd6* ^+/+^ but not *Cd6* ^-/-^ OT-II cells. These results would agree with previous reports of a positive role for CD6-CD166/ALCAM interaction in IS formation and stabilisation (11, 12). Further Western blot analysis of OVA-induced T-BMDC conjugates revealed attenuation of TCR signalling in *Cd6* ^-/-^ OT-II T cells compared to *Cd6* ^+/+^ OT-II ones, as evidenced by lower phosphorylation levels of CD3ζ-Y^83^, PLCγ-Y^783^ and ERK 1/2-T^202^/Y^204^, (**Figure 7B**), compatible with a co-stimulatory role for CD6 in early steps of T cell activation.

**Figure 8:**
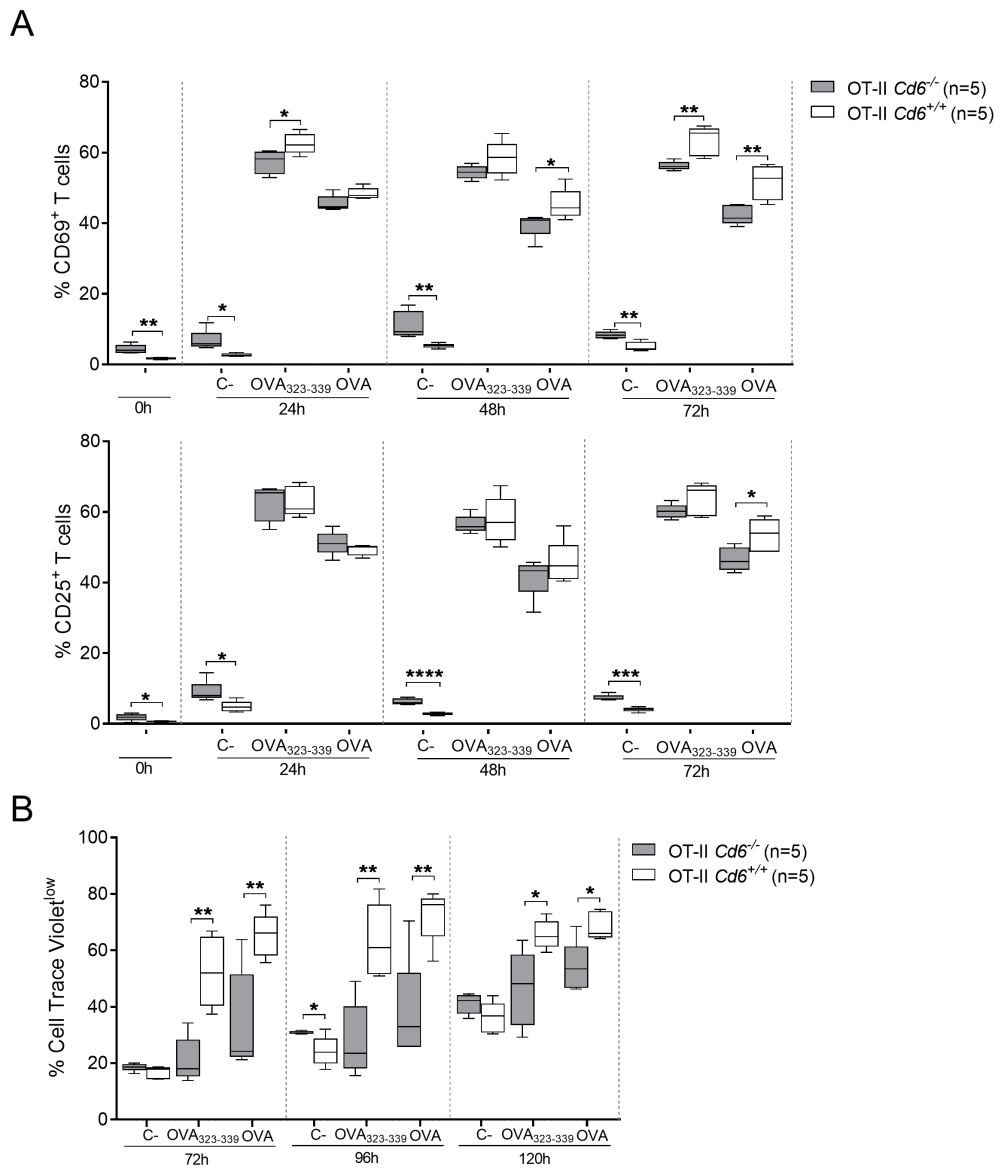
CD6 deficiency negatively regulates T cell activation marker expression and proliferative responses upon OVA-specific T-APC conjugate formation. **(A)** Percent of CD25^+^ (top) or CD69^+^ (bottom) T cells at different time points after conjugate formation of OVA_323-339_-loaded BMDCs with *Cd6^-/-^* or *Cd6^+/+^* OT-II CD4^+^ T cells. Unloaded (C-) and whole OVA protein-loaded BMDCs were included as negative and positive controls, respectively. A representative experiment out of three performed is shown. Data are presented as box and whiskers. Statistical differences were assessed by unpaired Student’s *t* test *p < 0.05; **p < 0.01; ***p < 0.001; ****p < 0.0001. **(B)** Percent of proliferating Cell Trace Violet^low^ T cells overtime from same cell conjugates as in **(A)**. A representative experiment out of three performed is shown. Statistical differences were assessed by unpaired Student’s *t* test *p < 0.05; **p < 0.01.

We show that *Cd6* ^-/-^ OT-II CD4^+^ T cells from OVA-specific T-BMDC conjugates reduce the percentage of CD69^+^ cells. This is concomitant with decreased CD25^+^ cells percentage at extended activation times with regard to *Cd6* ^+/+^ OT-II T cells (**Figure 8A**). Interestingly, unstimulated (vehicle) *Cd6* ^-/-^ OT-II cells exhibited higher percentages of cells positive for both activation markers, indicative of higher basal activation. Moreover, cell proliferation analyses using Cell Trace Violet also revealed a lower proliferative response for *Cd6* ^-/-^ OT-II T cells compared to *Cd6* ^+/+^ OT-II (**Figure 8B**). Taken together these *in vitro* findings support a role for CD6 as a positive modulator of TCR signalling upon antigen-specific T-APC interactions, thus promoting IS formation and subsequent T cell activation and proliferation events, as previously reported (11, 12).

## Discussion

CD6 is currently considered as a potential immunotherapeutic target in autoimmunity and cancer (30). However, the precise role of CD6 in lymphocyte activation remains unclear, with evidence of positive or negative regulatory functions depending on the experimental system, most often involving reductionist and/or artificial supraphysiological cell stimulation conditions (31). To shed light on this regard, a re-examination of CD6’s immunomodulatory properties under antigen-specific stimulation conditions was undertaken using wild-type and CD6-deficient OT-II mice (*Cd6* ^+/+^ and *Cd6* ^-/-^ OT-II). The results of this study, both *in vitro* and *in vivo*, provide new insights into the function of this multifaceted receptor.

OVA antigen-specific stimulation of total *Cd6* ^+/+^ and *Cd6* ^-/-^ OT-II splenocytes *ex vivo* enabled the study of T-cell responses in a complex cellular environment mimicking secondary lymphoid organs. This approach revealed that CD6 deficiency leads to enhanced CD4^+^ T-cell responses, as evidenced by increased *i*) expression of CD69 and CD25 activation markers, *ii*) cell proliferation, *iii*) IL-2 and IFN-γ cytokine production, and *iv*) *Pdl1*, *Fas* and *Fasl* mRNA expression, without significant changes in apoptosis upon a first challenge. These findings strongly support negative regulator of downstream TCR/CD3 signalling role for CD6 in antigen-specific lymphocyte activation. This aligns with Henriques et al. (2022) (32), who identified a phosphorylation-dependent sequence in CD6’s cytoplasmic tail that recruits inhibitory adaptors like UBASH3A, dampening TCR signalling and supporting a negative modulatory role for CD6 under certain conditions. Interestingly, CD6-deficiency did not result in a clear over-reactive phenotype following suboptimal mAb-induced CD3 crosslinking conditions. This indicates that CD3 crosslinking does not mimic T cell signalling following antigen-specific recognition, and that its signal strength overshadows the fine-tuning regulatory properties of CD6.

The OVA-induced DTH model provides further support for our *ex vivo* observations, demonstrating that CD6 deficiency results in an exacerbated inflammatory response to repeated antigen challenge. Key findings in *Cd6^-/-^* OT-II mice included increased *i*) ear thickness with associated oedema, and *ii*) ratios of Th1/Th17 and Th1/Th2 transcription factor mRNA levels in draining lymph nodes. These results align with previous reports of exacerbated CIA and cGvHD-induced lupus-like disease, as well as antiviral humoral responses in CD6-deficient mice (22, 25, 26) but contradict those reporting attenuated EAE, autoimmune uveitis and imiquimod-induced psoriasis disease models (3, 21, 24). The underlying reasons for these contradictory outcomes, such as potential variations in the genetic backgrounds of CD6-deficient mice (e.g., disease susceptible or resistant strains) and/or specific disease-inducing conditions (e.g., adjuvancy need, antigen recognition affinity, specific organ targeting conditions, physiopathological disease induction mechanism or immunisation schedules) remain a subject of speculation and require further investigations.

Our antigen-specific T-APC conjugate studies involving *Cd6* ^-/-^ or *Cd6* ^+/+^ OT-II CD4^+^ T cells and OVA-loaded BMDCs were consistent with earlier reports. Consequently, CD6 accumulates at the central supramolecular activation cluster (cSMAC) of the IS, where it associates with the TCR/CD3 complex (11). Recent work by Santos et al. (2024) (33) further demonstrates that CD6 translocation to the IS can occur independently of CD166/ALCAM engagement, suggesting additional mechanisms involving intrinsic cytoskeletal dynamics may also contribute to CD6 recruitment.

Furthermore, results showed maximised MTOC translocation to the proximity of the IS in CD4^+^ T cells from *Cd6* ^+/+^ *vs*. *Cd6* ^-/-^ OT-II mice, thus supporting the view that CD6-CD166/ALCAM interaction is crucial for proper IS formation and maturation (12). This finding was further validated by the reduced MTOC translocation in *Cd6* ^+/+^ OT-II CD4^+^ T-BMDC conjugates in the presence of rshCD6 protein, which competes with endogenous protein for its ligands. Western blot analysis revealed a trend towards lower phosphorylation of key early signalling molecules (CD3ζ-Y^83^, PLCγ-Y^783^ and ERK 1/2-T^202^/Y^204^) from *Cd6* ^-/-^ OT-II CD4^+^ T cells, advocating for a positive modulator of IS formation of CD6 plays in lymphocyte activation. This time-dependent co-localisation, as shown by Meddens et al. (2018) (34), underscores CD6’s role in stabilising TCR microclusters during IS maturation and further supports its involvement in modulating T cell activation. The amplification of TCR intracellular signalling by CD6 was further substantiated by the reduced surface expression of CD25 and CD69 activation markers and proliferation in CD4^+^ T-APC cell conjugates from *Cd6* ^-/-^ OT-II mice. Collectively, these observations point to a role for CD6 as an enhancer of antigen-specific T cell activation *in vitro*, as stated in previous studies using superantigens (11, 12).

The present findings reveal the dual nature of CD6 function in different OVA antigen-specific experimental settings *in vitro* (total splenocytes *vs*. T-APC heteroconjugates). The presence of CD6 favours IS formation through heterotypic T-APC adhesive contacts with CD166/ALCAM, while simultaneously facilitating activation signals generated by the TCR complex. The observed discrepancies in CD6’s fine-tuning function with regard to total splenocyte studies can be attributed to the complexity of the experimental systems used rather than the specific stimulation conditions used (OVA antigen *vs.* superantigens or CD3 crosslinking). *In vitro* studies using total splenocytes more closely resemble the intricate microenvironment of secondary lymphoid organs, encompassing various presenting cells (e.g., macrophages, dendritic cells, B cells) and responding T cells (e.g., helper, cytotoxic, regulatory) at different stages of maturation and activation (e.g., naïve, effector, memory). Moreover, these cells are influenced by other lymphoid e.g., NK, NKT, Tγδ), myeloid (e.g.,neutrophils, mast cells) and stromal (e.g., fibroblasts) cells which provide additional soluble factors or cellular contacts.

CD6 functions as a signal transducer, connecting extracellular and intracellular events. Its interaction with CD166/ALCAM is crucial but expression levels of these molecules in different experimental models can significantly impact lymphocyte activation (35). Additionally, the presence of other ubiquitous CD6 ligands such as Galectins 1 and 3 are known to influence adhesive CD6-CD166/ALCAM interactions and activation-induced T cell apoptosis, thereby making more complex the regulatory landscape (6, 36). In this regard, Galectin 1 acts as an immunoregulatory molecule itself or by binding to other receptor molecules on activated T cells, like CD69, attenuating T cell activation and Th1/Th17 responses (37–39). Understanding T cell behaviour at the population level provides valuable insights into their function in various contexts, including infectious, autoimmune, and tumour microenvironments. The concept of “quorum sensing” suggests that T cells may regulate their behaviour based on cell abundance, density, and activation state, allowing the population to act as a coordinated entity (40).

The CD6 receptor has been shown to operate as a multitask signalosome with opposite functions in T cell activation (41). Thus, under certain stimulation conditions (e.g., total splenocyte cultures) CD6 could downregulate TCR signalling by preferentially recruiting and interacting with inhibitory signalling effectors like UBASH3A, SHIP1 and RasGAP (41). This attenuation would prevent AICD events while enabling controlled lymphocyte responses. Conversely, reductionist T-APC conjugate experimental systems would enable CD6 interactions with activating signalling effectors like SLP-76, ZAP-70 and VAV1, resulting in enhanced lymphocyte activation. CD6 itself could also downregulate the expression of UBASH3A, a suppressor of TCR signalling, as reported recently in a mouse model of coronavirus infection (26).

In conclusion, we confirm that CD6 plays either an amplifying or attenuating role depending on the experimental context. This “dual” behaviour of CD6 as a rheostat-type signalosome highlights its potential as a target for developing therapeutic strategies in cancer, infectious diseases, and autoimmunity. Further research is warranted to fully elucidate the complex mechanisms governing CD6 function in different immunological contexts.

## Data Availability

The raw data supporting the conclusions of this article will be made available by the authors, without undue reservation.
